# Development and preliminary clinical feasibility of a Delphi-based aerobic exercise prescription for children with asthma

**DOI:** 10.3389/fped.2025.1700569

**Published:** 2025-12-09

**Authors:** Ying Zhang, Ze Kong, Kaixuan Yang, Zhiqiong Li, Ziye Yu, Longyan Liu, Chenxiao Meng, Jie Zhang

**Affiliations:** 1 Baoding Hospital of Beijing Children's Hospital, Capital Medical University, Baoding, China; 2 Capital University of Physical Education and Sports, Beijing, China

**Keywords:** asthmatic children, Delphi method, qualitative research, aerobic exercise prescription, evaluation

## Abstract

**Objective:**

This study aims to develop a clinically applicable aerobic exercise prescription for children with asthma using a two-round Delphi consensus process and to preliminarily evaluate its feasibility and safety of this prescription in a pre–post pilot study. The secondary goal was to explore preliminary signals of changes in exercise capacity, pulmonary function, and asthma control among asthmatic children after implementing the prescription—with consideration of potential confounding factors such as concurrent medication use and inhaler technique retraining—thereby providing preliminary evidence for non-pharmacological interventions in pediatric asthma management.

**Methods:**

Semi-structured interviews were conducted with 10 parents of asthmatic children, 10 medical professionals, and 10 sports rehabilitation experts to develop an initial aerobic exercise prescription based on the FITT (frequency, intensity, time, type) principle. Subsequently, this initial prescription was iteratively refined through two rounds of Delphi consultation involving 17 experts (specializing in pediatric pulmonology, pediatric nursing, and exercise rehabilitation). A consensus threshold of ≥75% agreement and authority metrics were predefined, and item-level expert feedback was used to modify the prescription. Finally, a 12-week prospective outpatient pilot study was conducted, including 15 asthmatic children aged 6–14 years who implemented the finalized prescription. Feasibility (recruitment rate, exercise adherence), safety (asthma-related adverse events), and clinical outcomes [6 min walk distance (6MWD), spirometry, Childhood Asthma Control Test (C-ACT)] were measured.

**Results:**

For the Delphi process, all 17 experts completed both rounds of consultation, with an effective response rate of 94.44% in Round 1 and 100% in Round 2. The expert authority coefficient (Cr) was 0.86, and the coefficient of variation (CV) for item importance scores decreased across rounds (from 0.07–0.16 in Round 1 to 0.07–0.13 in Round 2), indicating high expert consensus. For the pilot study, the recruitment rate reached 100%, and the average weekly exercise adherence was 2.7 ± 0.3 sessions. The incidence of asthma-related adverse events was 13.3% (mild wheezing and coughing), which did not interrupt training. Post-intervention outcomes showed significant improvements: 6MWD increased from 510.07 ± 31.96 to 539.27 ± 44.52 m (*P* < 0.001); forced expiratory volume in 1 s (FEV1)% predicted increased from 76.87 ± 5.63 to 87.61 ± 7.24 (*P* < 0.001); and C-ACT score increased from 17.60 ± 1.76 to 22.13 ± 1.46 (*P* < 0.001).

**Conclusions:**

The aerobic exercise prescription developed via the Delphi method is feasible and safe for short-term application in children with asthma, with preliminary signals of potential improvements in exercise capacity, pulmonary function, and asthma control. Notably, these observations may be influenced by confounding factors, including concurrent asthma medication adherence and standardized inhaler technique retraining. Due to the small sample size and uncontrolled pre–post design of the pilot study, definitive conclusions on efficacy or independent intervention effects cannot be drawn.

## 1 Introduction

Asthma is a chronic disease characterized by recurrent episodes of wheezing, coughing, shortness of breath, and chest tightness, typically accompanied by varying degrees of airflow obstruction and bronchial (airway) hyperresponsiveness (BHR or AHR) (1). Its etiology is complex, involving a combination of genetic factors, environmental influences, and abnormal immune system development in early life. Additionally, the prevalence and triggering factors of asthma exhibit significant variations across different periods and geographical regions (2). Asthma affects both adults and children, with a higher prevalence observed in children due to their weaker immune systems and lower resistance to infections (3, 4). As the most common chronic respiratory disease in childhood, asthma is typically characterized by recurrent episodes of wheezing, coughing, shortness of breath, and chest tightness, with symptoms often worsening or occurring more frequently at night and in the early morning (3, 4). Recurrent asthma attacks not only impair children's physical health and growth but also reduce their quality of life, placing a significant economic burden on families and society (5, 6).

According to the Third National Epidemiological Survey of Asthma in Urban Children in China, the overall prevalence of childhood asthma in major Chinese urban areas was 3.02%, with a 2-year current prevalence of 2.32% (7). In recent years, the global incidence of childhood asthma has shown a marked upward trend. Statistics from the Global Initiative for Asthma (GINA) estimate that approximately 300 million children worldwide are currently affected by asthma. The prevalence varies substantially across countries, ranging from 1% to 16% (8).

Over the past 25 years, although asthma-related mortality has significantly declined, no therapeutic approach has yet been able to completely cure this disease (9). Currently, pharmacotherapy remains the primary treatment for bronchial asthma worldwide. However, the treatment duration is typically prolonged, and adverse effects are often unavoidable. Moreover, even with regular medication, many children fail to achieve optimal asthma control (10). Some researchers suggest that early intervention with inhaled corticosteroids (ICS) in childhood asthma may improve long-term outcomes (11).

However, others report that while such treatment enhances symptom control in school-aged children with asthma, its benefits diminish within months after discontinuation (12). Consequently, researchers have proposed combining exercise therapy with conventional pharmacological treatment. Studies demonstrate that structured exercise programs can safely improve disease control in children with asthma. Furthermore, evidence indicates that regular physical activity reduces the risk of asthma exacerbations, enhances symptom management, and improves quality of life (13, 14).

Childhood represents a critical period for growth and development, during which both physical development and mental health are contingent upon regular physical exercise. The GINA report indicates that children with well-controlled asthma face a significantly reduced risk of experiencing coughing and wheezing symptoms following exercise. Consequently, it is recommended that asthmatic children engage in organized physical exercise. Exercise constitutes an essential component of children's growth, contributing to both physical and mental well-being.

Previous studies have demonstrated that regular exercise under scientific guidance can effectively assist asthmatic children in managing their condition, particularly by reducing nocturnal symptoms and promoting the recovery of vital capacity and other pulmonary function indicators (15). Meanwhile, regular exercise can significantly improve the quality of life of children with asthma, reduce mental health problems, and enhance their social skills. As children with asthma persist in participating in physical exercise, they will gradually build up self-confidence and develop a positive and self-disciplined attitude and lifestyle—benefits that may last a lifetime. The Delphi method (DM), also known as the expert survey method, involves consulting authoritative and representative experts in relevant fields. Based on the experts' feedback, the scheme is continuously revised until a consensus is reached (16). This study aims to adopt the Delphi expert consultation method to formulate an aerobic exercise prescription intervention program for children with asthma. In addition, this study will conduct a preliminary evaluation of the intervention effect, aiming to provide a reference for clinical medical staff in implementing exercise training for children with asthma.

## 2 Methods and participants

### 2.1 Study design

This study adopts a mixed-methods design, integrating qualitative research, the Delphi expert consultation method, and a preliminary clinical pilot study. During the qualitative research phase, semi-structured interviews were conducted with 10 parents of children with asthma, 10 medical professionals, and 10 sports rehabilitation experts. The interview data obtained were analyzed using systematic text condensation to develop an initial aerobic exercise prescription. Subsequently, two rounds of Delphi consultations were conducted, involving 17 experts in the fields of pediatric asthma, respiratory care, and sports medicine, to optimize the exercise prescription. The final prescription was evaluated through a pre–post controlled clinical trial, which included 15 children with asthma. Core indicators such as exercise capacity [6 min walk distance (6MWD)], lung function, and asthma control were observed. This study has been approved by the Ethics Committee of Beijing Children's Hospital Baoding Hospital, Affiliated to Capital Medical University (Approval Number: [2023(年)伦审【科】第(70)号]), and informed consent has been obtained from the guardians of all participating children.

### 2.2 Participants

The study subjects were recruited from the pediatric asthma clinic of the Baoding Branch of Beijing Children's Hospital Affiliated to Capital Medical University between June and December 2023. This clinic was selected as the sampling site because it serves as a regional core institution for pediatric asthma diagnosis and treatment, with stable patient sources and complete medical record data, which facilitates data collection and follow-up management for this pilot study. All 15 children with asthma included in this study were convenience samples from the aforementioned pediatric asthma clinic (not limited to inpatients); interventions were conducted under outpatient guidance and completed at home. It should be noted that convenience sampling may lead to potential selection bias, and the representativeness of the sample may be limited when extrapolating results to other regions or medical institutions.

The inclusion criteria were formulated in accordance with the asthma diagnostic criteria established by the GINA, which include the following: (1) aged 6–14 years; (2) confirmed diagnosis of asthma; (3) having used asthma medications (including β2-agonists, glucocorticoids, leukotriene receptor antagonists, or combination preparations of long-acting β2-agonists and glucocorticoids) within the past month; (4) having experienced symptoms such as dyspnea, chest tightness, or wheezing within the past month; and (5) presence of reversible airflow limitation, i.e., a ≥10% improvement in forced expiratory volume in 1 s (FEV1) 15 min after inhalation of 0.2 mg/kg (maximum 0.8 mg) salbutamol. A total of 15 children with asthma were finally included in the study. This sample size was determined with reference to (2), which recommended a sample size of 10–20 cases for pilot studies on pediatric asthma interventions. The 15-case sample is sufficient to verify the core objectives of this pilot study, including feasibility (e.g., recruitment completion rate and exercise adherence), safety (e.g., adverse event rate), and preliminary effectiveness (e.g., trends in lung function indicators). Additionally, this sample size can provide basic data (such as the variability of 6 min walk distance) for calculating the sample size of subsequent large-scale randomized controlled trials.

The exclusion criteria included (1) concurrent other respiratory system diseases; (2) concurrent autoimmune diseases or use of immunosuppressants; (3) concurrent acute or chronic systemic or local infections; and (4) participation in other clinical research trials. Two children withdrew from the study before the intervention began, with no reported reasons for withdrawal.

The inclusion criteria did not exclude children with poorly controlled asthma [Childhood Asthma Control Test (C-ACT) score ≤19]; however, there were no such children in the final sample. For the consideration of intervention safety, only children with a C-ACT score ≥20 were actually included, and 15 children were finally confirmed to participate in the study. This sample size (15 cases with well or partially controlled asthma) is in line with the sample size range for safety assessment of pediatric asthma exercise interventions proposed in (4), which ensures that potential exercise-related adverse events [such as exercise-induced bronchoconstriction (EIB)] can be initially identified while controlling the overall risk of the pilot study.

### 2.3 Methods

This study employed a mixed-methods approach, incorporating qualitative research, the DM, and a preliminary clinical pilot study. The DM (17, 18), also known as the expert consultation method, was developed by the RAND Corporation in the 1950s. It is an effective tool for collecting expert opinions. Through well-designed questionnaires distributed to expert panels via communication channels, this method gathers broad feedback and suggestions. After multiple rounds of consultation, the diverse and dispersed views of experts are gradually integrated to form a collective consensus, making it a commonly used decision-making technique.

Expert selection criteria: Experts were purposively selected based on predefined quantitative criteria to ensure a high level of expertise and representation from key relevant fields. The selection criteria included (1) a minimum of 10 years of professional experience in pediatric respiratory medicine, pediatric nursing, or exercise rehabilitation science; (2) a professional title at the intermediate level or above (e.g., attending physician, 主管护师, and lecturer); and (3) demonstrated scholarly contribution, with preference given to experts having an H-index ≥5 or having published at least 5 papers in relevant fields, as identified through databases such as China National Knowledge Infrastructure (CNKI) or Web of Science. A total of 20 experts were initially invited, and 17 who met the criteria and agreed to participate were finally included in the panel.

Content validity assessment: The content validity of the initial questionnaire was quantitatively assessed before the first round of Delphi consultation. A panel of five experts (not participants in the main Delphi study), comprising two pediatric pulmonologists, one pediatric nurse, and two sports medicine professors, evaluated each item of the draft questionnaire for relevance and clarity using a 4-point Likert scale (1 = not relevant/clear, 4 = highly relevant/clear). The Item-Level Content Validity Index (I-CVI) and Scale-Level Content Validity Index (S-CVI) were calculated. All items achieved an I-CVI of 0.80 or higher, and the S-CVI/Average was 0.92, indicating excellent content validity of the initial questionnaire.

Two rounds of expert consultation were conducted anonymously, with questionnaires distributed via email or WeChat. Initially, semi-structured interviews were conducted with parents of children with asthma, healthcare professionals, and exercise rehabilitation experts to identify core issues and needs in asthma management (19). Based on these findings, an aerobic exercise prescription was developed. Subsequently, the DM was used to invite experts for multiple rounds of consultation to reach consensus and refine the prescription. Finally, a preliminary clinical pilot study was conducted with 15 children with asthma to evaluate the feasibility, safety, and preliminary effectiveness of the prescription. Primary outcome measures included exercise capacity (6 min walk distance), pulmonary function, and asthma control (20, 21).

### 2.4 Exercise intervention

This study employed a standardized aerobic exercise prescription specifically designed for children with asthma aged 6–14 years. The prescription was developed based on qualitative research and the Delphi expert consultation method and strictly constructed in accordance with the FITT principle (frequency, intensity, time, type). The specific content is as follows:

• Frequency: Stratified according to asthma control status—3–5 times a week for children with well-controlled asthma (C-ACT ≥23 points); 4–6 times a week for children with partially controlled asthma (C-ACT 20–22 points) (adopting a “short-duration and multiple-session” mode to reduce single exercise load).

• Intensity: Dual evaluation criteria of heart rate and subjective feeling—moderate-to-high intensity for well-controlled children (exercise heart rate maintained at 60%–75% of maximum heart rate, with maximum heart rate calculated as 220 − age; subjective feeling as “slightly rapid breathing but able to speak normally”); moderate-to-low intensity for partially controlled children (exercise heart rate ≤60% of maximum heart rate, subjective feeling as “stable breathing and able to speak easily”), with real-time tracking via portable heart rate monitors.

• Time: The total duration of a single exercise includes three phases: warm-up phase (5–8 min, such as animal imitation exercises, slow rope skipping), training phase (20–30 min for well-controlled children; 2–3 sessions for partially controlled children, 5–10 min per session with 2–3 min of intermittent rest), and cool-down phase (5 min, such as slow walking, deep breathing training).

• Type: Priority is given to low airway irritation and high-interest sports—jogging, swimming (meeting the “asthma-friendly swimming pool standards”: chlorine concentration ≤0.5 ppm, water temperature 28℃–30℃), and cycling for well-controlled children; non-competitive swimming, walking, yoga, and breathing exercises for partially controlled children, avoiding high-intensity interval sports such as sprinting and fast rope skipping.

The goal of the intervention was to enhance participants' exercise capacity, lung function, and asthma control. The aerobic training was tailored to each child's physical fitness level (e.g., baseline value of 6 min walk distance) and asthma severity (GINA classification), aiming to improve their overall health and better manage asthma symptoms. The feasibility, safety, and effectiveness of the prescription were evaluated through a clinical trial. Outcome measures included 6MWD, pulmonary function, and asthma control, assessed both before and after the intervention (22).

### 2.5 Exercise monitoring and management procedures

1. Exercise intensity tracking: Adopting a dual model of “heart rate monitoring + subjective rating”—each child is equipped with a pediatric-specific heart rate monitor provided by the hospital (with marked applicability certification) to record real-time heart rate during exercise and ensure it remains within the target range; at the same time, the “Children's Rating of Perceived Exertion Scale (CR-10)” is used, where children self-rate every 5 min during exercise (1–3 points, easy; 4–6 points, moderate; 7–10 points, exhausted). When the score is ≥8 points or the heart rate exceeds the target range, the intensity is immediately reduced, or exercise is paused.
2. Safety assurance measures: (1) Pre-exercise assessment measures peak expiratory flow (PEF) 15 min before each exercise. If the PEF value is <80% of the predicted value, salbutamol (0.2 mg/kg, maximum dose 0.8 mg) must be inhaled first, and exercise can only start after re-measuring PEF to above 80% of the predicted value 30 min later. (2) Adverse event management: If symptoms of EIB such as coughing, wheezing, or chest tightness occur during exercise, or discomfort such as dizziness or nausea, exercise is immediately stopped, and the child is placed in a sitting position to rest. Emergency medications (such as salbutamol aerosol) are used if necessary, and the event time, symptoms, treatment measures, and outcome are recorded simultaneously. (3) Environmental control: Avoid exercise in environments with PM2.5 > 75 μg/m^3^, temperature <10 ℃, or humidity >80%. Indoor exercise must ensure good ventilation and avoid exposure to allergens such as dust mites and pollen.
3. Supervision responsibility division: (1) Medical professionals evaluate the exercise log (including exercise frequency, duration, intensity, and adverse events) through outpatient visits or remote follow-up (WeChat/phone) every 2 weeks and adjust the prescription based on lung function (FEV1% predicted) and C-ACT score. For example, the exercise duration of well-controlled children can be increased by 2–3 min per week (not exceeding the upper limit of 30 min). (2) Parents/guardians are responsible for daily exercise supervision, recording exercise logs (using standardized forms provided by the hospital), observing the child's response during exercise in real time, communicating with the medical team in a timely manner if adverse events occur, and assisting in completing warm-up and cool-down phase training to ensure standardized exercise procedures.

### 2.6 Statistical analysis

#### 2.6.1 Data entry and editing

Data entry and editing were performed using EpiData 3.1, and statistical analyses were conducted with SPSS 26.0. First, the Shapiro–Wilk test was used to assess the normality of all continuous variables (e.g., 6 min walk distance, FEV1% predicted, and C-ACT score). Only variables that conformed to a normal distribution (Shapiro–Wilk test, *P* > 0.05) were expressed as mean ± standard deviation (*x¯* ± *s*). For normally distributed data, paired *t*-tests were used to compare pre- and post-intervention data. To quantify the magnitude of intervention effects (i.e., the practical significance of pre–post differences), Cohen's *d* with 95% confidence intervals (95% CI) was calculated as the effect size. The interpretation of Cohen’s *d* followed conventional thresholds: *d* < 0.2, trivial effect; 0.2 ≤ *d* < 0.5, small effect; 0.5 ≤ *d* < 0.8, moderate effect; and *d* ≥ 0.8, large effect. Statistical significance was set at a two-tailed *P*-value of <0.05. No multiplicity adjustment was applied due to the exploratory feasibility nature of this study.

## 3 Results

### 3.1 General information of interviewees

This study interviewed 10 parents of children with asthma, labeled P1 to P10. Among them, three were male and seven were female. The participants ranged in age from 29 to 42 years, and five of them held a bachelor's degree or higher. Detailed demographic information of the interviewed parents is presented in Table 1.

**Table 1:** General information of parents of children with asthma.

Number	Relationship with the child	Age (years)	Educational level	Occupation	Child's age (years)	Child's gender
P1	Mother	38	Bachelor	Teacher	8	Male
P2	Father	33	Bachelor	Engineer	7	Male
P3	Mother	38	Associate degree	Pharmacist	11	Female
P4	Mother	36	Master	Teacher	9	Male
P5	Father	37	Senior high school	Self-employed	12	Female
P6	Mother	30	Junior high school	Self-employed	6	Male
P7	Mother	42	Bachelor	Civil servant	14	Female
P8	Father	38	Bachelor	Teacher	9	Male
P9	Mother	37	Senior high school	Freelancer	12	Male
P10	Mother	29	Junior high school	Self-employed	6	Male

### 3.2 General information of respiratory medical staff and sports rehabilitation experts

This study interviewed five pediatric pulmonologists, two pediatric respiratory nurses, and three exercise rehabilitation specialists (coded as B1–B10), including three males and seven females. The participants ranged in age from 36 to 59 years. Among them, four experts had over 20 years of work experience. Six held senior or associate senior professional titles, and three had a master's degree or higher. Detailed information about the interviewees is presented in Table 2.

**Table 2:** General information of pediatric respiratory medical staff and exercise rehabilitation experts.

Number	Age (years)	Gender	Professional title	Position	Work experience (years)	Educational background
B1	50	Female	Chief physician	Department director	28	Bachelor
B2	43	Male	Chief physician	None	17	Bachelor
B3	42	Male	Chief physician	None	15	Bachelor
B4	36	Female	Chief physician	None	11	Bachelor
B5	39	Female	Associate chief physician	Department deputy director	15	Bachelor
B6	37	Female	Chief physician	None	13	Bachelor
B7	53	Female	Associate chief physician	Head nurse	33	Bachelor
B8	48	Male	Professor	None	24	Doctor
B9	45	Female	Associate professor	None	19	Doctor
B10	59	Female	Professor	None	36	Master

### 3.3 Draft of exercise program formulation

Based on the interview findings, relevant literature, and internal discussions within the research team, a preliminary exercise prescription was developed for children with asthma. The prescription was formulated by considering the level of asthma control, previous exercise experience, and results from individual physical fitness assessments. In addition, factors such as the child's age, psychological characteristics, family environment, and personal interests were taken into account.

In line with the principle of gradual progression, appropriate exercise goals were set, a sustainable prescription was proposed, and follow-up plans were outlined. According to the GINA 2023 guidelines and the revised C-ACT scale by Wu (19), well-controlled asthma is defined as a C-ACT score ≥23, while partially controlled asthma corresponds to a score of 20–22. Details are presented in Table 3.

**Table 3:** Draft of the exercise prescription.

Asthma control status	Exercise prescription recommendations	Precautions
Well-controlled asthma	Exercise type: Based on the child's interests, jogging, walking, cycling, etc. Swimming is preferred if conditions permit.	Avoid exposure to allergens and environmental irritants; perform warm-up exercises, and use preventive medication before exercise if necessary; suspend exercise when suffering from respiratory infections; start with an appropriate amount of exercise and gradually achieve the exercise goal
	Exercise intensity: Moderate-to-high intensity is recommended.	
	Exercise duration and frequency: Each exercise session lasts 20–30 min (including 5 min warm-up and 5 min cool-down); target frequency is 3–5 days per week (i.e., 3–5 sessions weekly, 1 session per day when exercising)	
Partial asthma control	Exercise type: Exercises with short physical output time (<5–10 min) and low respiratory load, such as non-competitive swimming, walking, jogging, yoga, and breathing exercises.	Avoid strenuous sports such as sprinting and fast rope skipping; avoid continuous physical output (≥10 min) such as long-distance running and long-distance swimming; avoid exercising in dry and cold environments; avoid exposure to allergens and environmental stimuli; start with an appropriate amount of exercise (intensity × time) and proceed gradually
	Exercise intensity: Moderate-to-low intensity is recommended (respiratory rate < twice the resting state, exercise heart rate <60% of maximum heart rate or <130–140 beats/min).	
	Exercise duration and frequency: Each exercise session lasts 5–10 min (including 3 min warm-up and 3 min cool-down); target frequency is 4–6 days per week, with 2–3 intermittent sessions per day (total weekly target: 8–12 sessions); exercise purpose is achieved by accumulating total weekly session time (target: ≥60 min/week)	

(1) Exercise adherence calculation: Adherence rate = (Actual number of completed exercise sessions/Planned weekly target sessions) × 100%. For well-controlled asthma, planned weekly target sessions = 3–5; for partially controlled asthma, planned weekly target sessions = 8–12. A session is considered “completed” if the actual exercise duration reaches ≥80% of the recommended single-session duration (e.g., ≥16 min for well-controlled asthma and ≥4 min for partially controlled asthma).

(2) Adjustment principle: If the adherence rate is <60% for 2 consecutive weeks, the research team will re-evaluate the child's exercise tolerance and adjust the prescription (e.g., reduce intensity, shorten single-session duration) in combination with C-ACT score and pulmonary function results.

### 3.4 Basic information of experts

A total of 17 experts participated in two rounds of Delphi consultation. All 17 experts completed both rounds of consultation without dropping out. In the first round, 18 questionnaires were distributed, and 17 valid ones were collected (effective response rate: 94.44%). In the second round, all 17 distributed questionnaires were returned and valid (effective response rate: 100%), which guaranteed the reliability of the consensus results. These experts included pediatric respiratory clinicians from Beijing Children's Hospital affiliated with Capital Medical University and Baoding Central Hospital, as well as clinical doctors from the Department of Sports Medicine at Capital University of Physical Education and Sports, asthma care specialists, and professors of physical education.

The 17 experts represented three core domains: pediatric pulmonology (9 experts, 52.94%, including 5 chief physicians), pediatric nursing (2 experts, 11.76%, including 1 associate chief nurse), and exercise rehabilitation (6 experts, 35.29%, including 2 professors and 1 associate professor). All experts had over 10 years of experience in their respective fields. Their research areas covered pediatric asthma, clinical nursing, early childhood physical education, and sports training. The participating experts possessed both extensive practical experience and strong theoretical foundations. Detailed expert information is presented in Table 4.

**Table 4:** General information of the experts.

Basic information		Number of respondents in two rounds of consultation	
		Details	Percentage (%)
Gender	Male	6	35.29
	Female	11	64.71
Age (years)	30–39	4	23.53
	40–49	8	47.06
Ethnicity	Han	17	100
	Other ethnicities	0	0
Highest educational qualification	Bachelor	11	64.71
Professional title	Master	2	11.76
	Doctor	4	23.53
	Senior	5	29.42
	Associate senior	6	35.29
Job type	Intermediate	6	35.26
	Clinical	11	64.71
	Teaching	6	35.29
Work experience (years)	10–19	9	52.94
Research direction	20–29	4	23.53
	Over 30	4	23.53
	Pediatric respiratory medicine	9	52.94
	Pediatric nursing	2	11.76
	Exercise rehabilitation	6	35.29

This study conducted two rounds of expert consultation. The level of expert participation was assessed based on the effective response rates of the two rounds of questionnaires. In the first round, 18 questionnaires were distributed and collected, with 17 deemed valid, resulting in a 100% response rate and a 94.44% effective response rate. In the second round, all 17 questionnaires were returned and considered valid, yielding both a 100% response and an effectiveness rate. In general, a response rate above 80% indicates high expert engagement. In this study, both rounds exceeded 90%, reflecting the experts’ strong enthusiasm and commitment to the consultation process.

The authority coefficient (Cr) of the experts was calculated based on two components: (1) the basis of judgment regarding the consultation content (Ca) and (2) the expert's self-assessed familiarity with the content (Cs). Familiarity was categorized as “very familiar,” “familiar,” “somewhat familiar,” or “unfamiliar,” with corresponding scores of 1.0, 0.75, 0.5, and 0.25, respectively. The basis of judgment included four dimensions: theoretical analysis, practical experience, peer knowledge, and subjective judgment. Experts rated the influence of each dimension as “high,” “moderate,” or “low,” with assigned weights as follows: theoretical analysis (0.5, 0.4, 0.3), practical experience (0.3, 0.2, 0. 1), peer knowledge (0.1, 0.1, 0. 1), and subjective judgment (0.1, 0.1, 0. 1).

SPSS 26.0 was used for statistical analysis. The results showed an average familiarity score (Cs) of 0.81 and a basis of judgment score (Ca) of 0.91. The authority coefficient was calculated as Cr = (Ca + Cs)/2 = (0.809 + 0.9118)/2 = 0.86, indicating a high level of expert authority.

### 3.5 Expert consultation results and feedback

In the first round of consultation, experts rated the importance of 22 items in the exercise prescription. The importance scores ranged from 4.24 to 5.00, with arithmetic means between 4.24 and 5.00. The full score frequency (percentage of experts giving the maximum score of 5) ranged from 35% to 92% across different items. The coefficients of variation (CVs) for these items ranged from 0.07 to 0.16. All CV values were ≤0.15, indicating a high degree of consistency among expert evaluations (Table 5).

**Table 5:** Recommendations from the first round of expert consultation.

Revision status	Expert recommendations for revision	Revision results
Addition	1. It is recommended to clarify the specific process and content of training	Add an introduction to the training process and content for relevant personnel, and refine the training division of labor among pediatric respiratory physicians, rehabilitation exercise experts, and parents of children
	2. It is recommended to add specific pre-exercise evaluation standards	Supplement pre-exercise evaluation standards: including ACT score, number of acute exacerbations in the past month, basic lung function (FEV1/FVC), and add the 6 min walking test to measure the child's exercise ability
	3. It is recommended to add a link for team members to establish trust with children and their parents	Add a link for team members to establish trust with children and their parents, including explaining the benefits of exercise, listening to family concerns, and signing safety commitments
	4. It is recommended to list specific criteria for pausing exercise to distinguish between exercise-induced asthma and simple fatigue	List specific criteria for pausing exercise according to expert recommendations, indicating that if the PEF value is <80% of the estimated value, it is necessary to inhale reliever drugs or participate in low-intensity activities for observation
	5. It is recommended to clarify the standards and plans for resuming exercise after pausing	Specify in detail the standards and plans for resuming exercise after pausing. Re-measure PEF 10–20 min after exercise is paused. If it returns to the normal value, exercise can be resumed normally
	6. It is recommended to clarify the purchase method of heart rate monitors	Clarify that heart rate monitors will be distributed to each child by the hospital, and mark the pediatric applicability certification
	7. It is recommended to add content about warm-up exercises	Supplement the content of warm-up exercises and add a “fun warm-up module,” such as 5 min animal imitation games (stretching) + 5 min slow rope skipping, taking into account children's interests and safety
	8. It is recommended to improve the follow-up content	Add the inspection and guidance of children's exercise training by team members after follow-up, focusing on recording exercise compliance and symptom changes
Revision	9. It is suggested that the implementation part of the exercise plan should specifically distinguish different age groups, including exercise forms, time, etc.	According to expert suggestions, specifically distinguish different age groups and design exercise plans by age stratification

In the second round of expert consultation, the average importance scores for the 23 items in the exercise prescription ranged from 4.53 to 5.00. The full score frequency improved to 47%–100% across items, indicating increased consensus on item importance. The CVs ranged from 0.07 to 0.13. All CV values were below 0.15, indicating a high level of agreement among the experts on the importance of each item.

In the second round of expert consultation, the average importance scores for the 23 items in the exercise prescription ranged from 4.53 to 5.00. The CVs ranged from 0.07 to 0.13. All CV values were below 0.15, indicating a high level of agreement among the experts on the importance of each item.

During this round, experts proposed several suggestions for adding or modifying specific items. After thorough discussion and analysis, the research team identified three key revisions. Table 6 presents the detailed content of these proposed changes.

**Table 6:** Recommendations from the second round of expert consultation.

Revision status	Expert recommendations for revision	Revision results
Addition	1. It is recommended that experts add explanations on the purpose and precautions of exercise during health education	According to the recommendation, experts add explanations on the purpose and precautions of exercise, and clarify the mechanism by which aerobic exercise improves lung function (such as enhancing diaphragm strength and reducing the level of inflammatory factors)
	2. The health education part should add guidance for parents or guardians to help them understand the content and implementation methods of the prescription, and assist and supervise their children in exercise in daily life	Add health education content for parents or guardians according to expert recommendations
Revision	3. Attention should be paid to the selection of swimming venues during implementation. At present, the disinfection methods of some swimming venues are not conducive to asthmatic patients	Pay attention to selecting swimming pools suitable for asthmatic patients, and formulate “asthma-friendly swimming pool screening standards”: chlorine concentration ≤0.5 ppm, equipped with an air circulation system, and providing warm water swimming pools (28℃–30℃)

Following these modifications, the research team finalized the exercise prescription and intervention plan for children with asthma, which consisted of 5 primary-level items, 13 secondary-level items, and 23 tertiary-level items, as detailed in Supplementary Appendexes B and C.

### 3.6 General information on children with asthma

A total of 15 children with asthma met the inclusion criteria for this study, including 9 boys and 6 girls. The mean age was 9.67 ± 1.84 years. The average number of acute asthma exacerbations in the past three months was 2.40 ± 0.83 episodes. Detailed demographic and clinical information of the participants is presented in Table 7.

**Table 7:** Demographic and clinical characteristics of children with asthma.

Basic information		Number (*n*)/mean $\left(\bar{x}\pm s\right)$	Proportion, *n* (%)
Gender	Male	9	60.0
	Female	6	40.0
Child's age (years)		8.67 ± 0.50	
Family member's age (years)		35.87 ± 1.06	
Place of residence	Urban	9	60.0
	Rural	6	40.0
Father's/mother's educational level	Junior high school	5	33.3
	Senior high school	8	53.3
	College and above	2	13.3
Family economic status	Below 5,000	3	20.0
	5,000–8,000	9	60.0
	Over 8,000	3	20.0
Medical expense payment method	Self-payment	3	20.0
	Medical insurance	12	80.0
Family history of asthma	None	7	46.7
	Yes	8	53.3
Duration since asthma onset	≤1 year	2	13.3
	2–3 years	6	40.0
	4–5 years	5	33.3
	>5 years	2	13.3
Asthma severity (GINA classification)	Mild intermittent	2	13.3
	Mild persistent	7	46.7
	Moderate persistent	5	33.3
	Severe persistent	1	6.7 (Note: This case is severe asthma as defined by GINA, requiring high-dose ICS, different from insufficient treatment)
Baseline inhaler technique assessment	Good mastery	8	53.3
Baseline medication adherence assessment	Poor mastery	7	46.7 (Note: Discovered during pre-intervention assessment, all received standardized retraining and regular checks during the study)
	Good adherence (≥80%)	9	60.0
	Poor adherence (<80%)	6	40.0 (Note: Discovered through questionnaire/pill count assessment before intervention, during the study, through education, reminders, and monitoring, all children's adherence improved, among which four cases improved to good level, and two cases improved but still <80%)

### 3.7 Feasibility analysis

This study planned to include 15 children with asthma, and 15 were successfully recruited, yielding a recruitment completion rate of 100%. On average, the children achieved their exercise goals 2.7 ± 0.3 times per week, indicating a high level of exercise adherence.

In addition, pulmonary function test data (FVC% predicted, FEV1% predicted, and FEV1/FVC%) and asthma control questionnaire responses were complete, with a data missing rate of 0%, demonstrating high protocol operability.

Furthermore, online and offline follow-up surveys showed high levels of acceptance of the aerobic exercise intervention among children and high satisfaction levels among their parents, indicating strong feasibility of the intervention.

### 3.8 Safety analysis

The incidence of asthma-related adverse events during the intervention period was 13.3%. Two children experienced mild wheezing accompanied by light coughing, which resolved after a short rest and did not affect the continuation of the intervention.

No serious adverse events occurred, and no exercise-related injuries were reported. All sessions were conducted under safety monitoring, and the exercise prescription was adjusted flexibly based on each child's tolerance. The intervention was proven to be safe and controllable, and parental feedback confirmed that the program had a good safety profile with a low incidence of adverse events.

### 3.9 Effectiveness analysis

**Exercise capacity comparison:** A paired sample *t*-test was used to compare 6MWD of children with asthma before and after the intervention. The results showed a significant improvement from 510.07 ± 31.96 m at baseline to 539.27 ± 44.52 m post-intervention (*t* = −5.21, *P* < 0.001), indicating that the exercise intervention effectively improved exercise endurance.
**Pulmonary function comparison:** Pulmonary function indices of the 15 participants were compared before and after the aerobic exercise intervention. All pulmonary function indicators showed varying degrees of improvement after the intervention. Analysis using the Wilcoxon signed-rank test showed that FEV1% predicted significantly increased from 76.87 ± 5.63 to 87.61 ± 7.24 (*Z* = −3.82, *P* < 0.001); forced vital capacity % predicted (FVC% predicted) slightly increased from 105.92 ± 12.69 to 109.79 ± 10.92 (*Z* = −2.35, *P* = 0.019); and FEV1/FVC% ratio significantly increased from 73.19 ± 6.74 to 80.27 ± 8.10 (*Z* = −3.94, *P* < 0.001) (Table 8). All indicators showed statistically significant differences before and after the intervention (*P* < 0.05), with the most significant improvements in FEV1% predicted and FEV1/FVC% (*P* < 0.001). These results indicated significant differences in all pulmonary function parameters before and after the intervention (*P* < 0.05), with the most notable improvements observed in FEV1% predicted and FEV1/FVC% (*P* < 0.001).

**Table 8:** Comparison of pulmonary function indicators before and after the intervention.

Group/Indicator	FEV1% predicted	FVC% predicted	FEV1/FVC%
Before intervention	76.87 ± 5.63	105.92 ± 12.69	73. 19 ± 6.74
After intervention	87.61 ± 7.24	109.79 ± 10.92	80.27 ± 8.10
Test statistic	*Z* = −3.82	*Z* = −2.35	*Z* = −3.94
*P*-value	0.000	0.019	0.000

#### 3.9.1 Comparison of asthma control scores

The C-ACT scores of children with asthma improved significantly over the intervention period. Analysis using the Wilcoxon signed-rank test showed that the score increased from 17.60 ± 1.76 before the intervention to 22.13 ± 1.46 after the intervention, with a statistically significant difference (*Z* = −3.98, *P* < 0.001), representing a 25.74% improvement.

## 4 Discussion

### 4.1 Reliability of expert consultation results

The 17 experts consulted in this study all held at least a bachelor's degree, with 23.53% holding a doctoral degree and 11.76% holding a master's degree. Their areas of expertise covered pediatric respiratory medicine, pediatric nursing, and exercise rehabilitation. All experts held intermediate-level or higher professional titles, with 29.42% holding senior titles and 35.29% associate senior titles. Their ages ranged from 36 to 61 years, and all had at least 10 years of professional experience in their respective fields. Among them, 23.53% had over 30 years of experience, and another 23.53% had 20–29 years of experience, demonstrating strong theoretical knowledge and practical experience, thereby ensuring the credibility of their evaluations and suggestions.

The effective response rate was 94.44% in the first round of questionnaires and 100% in the second, with both rates exceeding 90%, indicating strong expert engagement. The authority coefficient (Cr) was 0.86, reflecting high expert authority and reliable prediction quality. Moreover, the CVs showed a decreasing trend between rounds, indicating increasing consensus among experts. Kendall's *W* tests for both rounds revealed *P*-values of <0.01, confirming that the consultation results were coordinated and reliable.

### 4.2 Innovation and applicability of the exercise prescription

Aerobic exercise has been widely recognized as a core non-pharmacological treatment for children with asthma. Several international studies (13–15) have demonstrated that aerobic exercise improves pulmonary function and quality of life in asthma patients and is generally safe for children with mild to moderate asthma. However, issues such as unclear intensity guidelines and the adult-centered design of existing prescriptions limit their application to children.

In this study, qualitative interviews revealed parental perceptions and needs regarding aerobic exercise, including concerns about asthma exacerbation and lack of professional guidance. A basic framework of the exercise prescription was initially developed and subsequently refined through two rounds of Delphi consultation, integrating feedback from pediatric respiratory and rehabilitation experts. This iterative process significantly improved the clinical applicability of the prescription.

### 4.3 Feasibility of the intervention plan

The personalized aerobic exercise prescription in this study enhanced children's engagement by facilitating active communication and dynamic adjustment of the training plan. The recruitment completion rate was 100%, and participants achieved their weekly exercise targets an average of 2.7 ± 0.3 times, indicating good adherence.

Complete pulmonary function test data (FVC% predicted, FEV1% predicted, FEV1/FVC%) and asthma control questionnaire responses demonstrated the high operability of the intervention. Follow-up surveys indicated that the flexibility and safety monitoring of the program led to good acceptance by children and high satisfaction among parents, although it should be noted that observed changes in clinical outcomes (e.g., C-ACT score) may reflect potential improvements from a combination of the exercise intervention, optimized medication adherence, and inhaler technique retraining (rather than the exercise alone).

Notably, the average weekly exercise adherence in this study was 2.7 ± 0.3 sessions, which was slightly lower than the predefined target of ≥3 sessions per week (consistent with the “3–5 days/week” frequency in the well-controlled asthma group of Table 3). *Post hoc* analysis of follow-up records identified three potential contributing factors:

1. Home-based implementation barriers: Most participants (60.0%, *n* = 9) lived in urban areas, but 40.0% (*n* = 6) resided in rural regions (Table 7). Rural families reported limited access to safe exercise spaces (e.g., dedicated parks and indoor gyms) and inconsistent supervision due to parental work schedules, which reduced the likelihood of completing daily sessions.
2. Asthma symptom variability: Although all children had a C-ACT score ≥20 (partially to well-controlled), four participants (26.7%) reported mild nighttime coughs during the intervention (not classified as adverse events). This symptom-related discomfort led to voluntary session cancellations, particularly in the first 4 weeks of the 12-week program.
3. Child engagement challenges: For children aged 6–8 years (*n* = 5, 33.3%), the initial exercise plan (e.g., 20 min jogging) lacked age-appropriate interactivity. Follow-up interviews with parents revealed that these younger children often lost interest after 10–15 min, resulting in incomplete sessions (not counted in adherence calculations per Table 3’s “≥80% duration” standard).

However, the study's small sample size and short duration limit its generalizability. In addition, potential confounding factors such as concurrent medication and improved inhaler technique were not controlled, so the observed improvements cannot be attributed solely to the intervention. Future studies should use randomized controlled trial (RCT) designs to isolate the effects of exercise.

### 4.4 Safety of the intervention

Given the particular vulnerabilities of children with asthma, safety is paramount in exercise interventions. This study monitored asthma-related adverse events to preliminarily assess the clinical safety of the prescription. All 15 children completed the 3-month intervention, and the adverse event rate was 13.3%, involving only mild coughing and wheezing that resolved with rest and did not disrupt training. No serious asthma-related incidents or exercise-related injuries were reported.

A combination of remote supervision and home-based training reduced the likelihood of errors, and timely communication between medical staff and families enabled rapid risk management. The intervention was safe and well-controlled. Nevertheless, the limited sample size and intervention period call for future studies with longer follow-up and optimized training intensities to further evaluate long-term safety.

### 4.5 Effectiveness of the intervention

This self-controlled study showed that 6MWD improved after the intervention, reflecting preliminary signals of enhanced exercise endurance. Pulmonary function parameters including FEV1% predicted, FVC% predicted, and FEV1/FVC% also exhibited positive changes—these observations, while promising, may reflect a combination of factors including the aerobic exercise prescription, sustained medication use, and improved inhaler technique (Table 7), rather than independent effects of exercise alone. Thus, we cannot definitively attribute these changes to the exercise intervention, nor can we confirm it as the sole driver of potential improvements in respiratory muscle endurance, ventilation efficiency, or airway expansion.

In addition, post-intervention C-ACT scores increased, reflecting either reduced asthma symptom frequency or improved self-management. However, due to the lack of a parallel control group and the continuation of routine care during the intervention, improvements may be influenced by medications, environmental factors, or other variables.

Given the small sample size and limited duration, the strength of the evidence is modest. Future studies should adopt well-designed RCTs comparing “aerobic exercise + standard care” with “standard care alone” and extend both sample size and intervention duration to verify long-term effectiveness.

### 4.6 Study limitations and future directions

Through qualitative interviews with 10 parents and 10 medical/rehabilitation professionals, this study identified the perceptions, needs, and challenges associated with aerobic rehabilitation for children with asthma. Together with expert input, this informed the theoretical foundation for a structured prescription.

A multi-round Delphi process refined the draft into a standardized aerobic exercise prescription with 5 primary domains, 13 secondary items, and 23 tertiary items covering multidisciplinary collaboration, health education, exercise training, follow-up management, and outcome evaluation. The results of the pilot clinical study involving 15 children suggest that aerobic exercise improved treatment adherence, asthma control, and 6MWD, with statistically significant differences (*P* < 0.05). Thus, the program appears feasible, safe, and effective, providing both theoretical and practical guidance for clinical implementation and broader promotion.

However, this study adopted a single-center design and included only children with asthma from Baoding Hospital of Beijing Children's Hospital (January–December 2024). As a regional core institution for pediatric asthma specialty care, this center differs from primary hospitals or non-specialized medical institutions in terms of diagnosis-treatment processes (e.g., frequency of standardized medication guidance and intensity of follow-up management) and patient baseline characteristics (e.g., parents' health literacy and family medical support capacity). Meanwhile, environmental factors in Baoding—such as urbanization level (e.g., urban green coverage), air quality (e.g., annual average PM2.5 concentration), and climatic conditions (e.g., humidity and temperature variation)—are significantly distinct from those in northern high-pollution regions, southern humid areas, or rural areas. These differences may lead to variations in children's tolerance to aerobic exercise and asthma symptom triggers. Such single-center-specific factors not only introduce selection bias but also hinder the extrapolation of the exercise prescription's effects (e.g., adherence and symptom improvement) to other regions or institutions with different clinical conditions. Particularly in rural areas or regions with limited medical resources, the prescription's adaptability requires further verification. In addition, the lack of a RCT design prevents us from isolating the effects of aerobic exercise from concurrent factors such as improved medication adherence or seasonal changes in asthma symptoms. The small sample size (*n* = 15) not only reduces statistical power—making it impossible to detect differences in subgroup analyses (e.g., age, 6–9 years vs 10–14 years; asthma severity: mild persistent vs moderate persistent)—but more critically fails to fully capture the individual heterogeneity of children with asthma (e.g., allergen sensitization status and baseline lung function). For instance, none of the 15 participants had a history of severe allergies, yet clinically, 20%–30% of asthmatic children have comorbid allergic rhinitis [e.g., Chinese Guidelines for the Diagnosis and Management of Childhood Asthma (2020 Edition)]. Such children are more sensitive to exercise environments, and our results cannot reflect their tolerance to the prescription. Additionally, the 12-week intervention duration limits assessments of long-term safety and sustained effectiveness, further weakening the generalizable value of the findings for long-term clinical application. Notably, this study only included children with a C-ACT score ≥20 (well-controlled or partially controlled asthma) due to safety concerns, with no poorly controlled cases (C-ACT ≤19) in the final sample. However, clinically, 30%–40% of asthmatic children have poorly controlled conditions (e.g., GINA 2023 Report). These children experience more frequent symptom attacks and lower exercise endurance, and their tolerance to exercise intensity/types differs significantly from well-controlled peers—for example, they may not tolerate the “moderate-to-high intensity” exercise recommended herein and require stricter intensity grading and safety monitoring. Our results only apply to well-controlled children and cannot cover the large clinical population of poorly controlled patients, creating a critical adaptation gap for broad prescription application. This limitation is more pronounced in primary medical institutions, where the proportion of poorly controlled children is higher. Furthermore, due to the small sample size (*n* = 15) in this study, the statistical method for comparing pre- and post-intervention data was adjusted from a parametric test (paired *t*-test) to a non-parametric test (Wilcoxon signed-rank test) to avoid the risk of invalid normality assumptions for small samples. However, limited by the sample size, random errors in statistical results cannot be completely excluded, and future large-sample studies can further verify the stability of the intervention effect. Meanwhile, as a pilot study, the determination of the sample size (*n* = 15) was mainly based on clinical feasibility (e.g., difficulty in recruiting outpatients and follow-up management load), without clear scientific basis through systematic literature review [e.g., sample size calculation formula based on the variability of the primary outcome indicator (6 min walk distance), expected effect size, *α* = 0.05, and *β* = 0.80], lacking a standardized sample size calculation process. The insufficient standardization in sample size determination, combined with limitations such as low statistical power and high risk of random error caused by the small sample size, collectively weakens the statistical reliability of the study results and the robustness of their interpretation. For example, the slight improvement in pulmonary function indicators (e.g., FVC% predicted) may be difficult to exclude the impact of chance factors due to insufficient sample size, making it impossible to clearly attribute the effect to the aerobic exercise prescription. Future research should target these generalizability limitations: (1) conduct multicenter studies involving institutions across regions (northern high-pollution cities, southern humid cities, rural areas) and levels (tertiary hospitals, secondary hospitals, community health centers), using stratified sampling to ensure subgroup proportions (e.g., poorly controlled children and those with comorbidities) match clinical reality. (2) Expand the sample size to at least 100 cases (referencing pediatric asthma intervention sample size standards, e.g., calculated based on FEV1% predicted variation) to enhance statistical power and support subgroup analyses. (3) Include children with poorly controlled asthma (C-ACT ≤19) and design stratified exercise plans (e.g., a “low-intensity initial phase” for this group) to verify prescription adaptability. (4) Integrate the “hospital–community–family” model with community-specific adaptations—such as “equipment-free exercise plans” for rural areas and “indoor alternative programs” for high-pollution regions—to improve clinical generalizability and accessibility.

## Data Availability

The datasets presented in this study can be found in online repositories. The names of the repository/repositories and accession number(s) can be found in the article/Supplementary Material.
