# Supplementary material for: Development and preliminary clinical feasibility of a Delphi-based aerobic exercise prescription for children with asthma
Source: Front Pediatr. 2025 Dec 9;13:1700569. doi: 10.3389/fped.2025.1700569 (PMC12722912; doi:10.3389/fped.2025.1700569)
Supplement: Supplementary file 1 [file Supplementaryfile1.docx]

Appendix A: Proposed Framework and Preliminary Intervention Plan for Exercise-Based Oxygen Therapy in Children with asthma

Round One: Expert Consultation Form

Dear Expert,

We sincerely invite you to serve as a consulting expert for this project, in recognition of your outstanding achievements and contributions in the fields of asthma, exercise science, and pediatrics. Your valuable suggestions will serve as a key basis for improving this program.

With the ongoing research on the effects of exercise interventions on children with asthma, we intend to explore how exercise-based oxygen therapy might impact the treatment outcomes of such children. By deepening our understanding of the mechanism and significance of exercise intervention in treating children with asthma, we aim to lay the groundwork for further research in this area.

This consultation form mainly focuses on the design and preliminary proposal for exercise-based oxygen therapy in children with asthma. We seek your professional opinion as a healthcare expert in this field, which will serve as a foundation for literature analysis and expert discussion in the next phase.

This round of consultation is the first, consisting of three parts: background information, general information, and the expert questionnaire. To ensure a more comprehensive and reasonable proposal, we may conduct 2–3 rounds of consultation. We would greatly appreciate your input and timely response within 10 days of receipt of this letter to facilitate the next phase of our work.

Thank you very much for your support and assistance! We wish you smooth work and good health.

**Part One: Expert Consultation Form**

Project Overview:

1.In the early stages, our research team conducted a literature review focusing on the effects of physical exercise combined with oxygen therapy on the prognosis of children with asthma. This provides strategic support for the practical implementation of this proposed intervention.

2.The target participants for the intervention are children with asthma who meet the inclusion criteria.

3.The intervention content and format: the intervention will be carried out in collaboration with hospitals affiliated with Capital Medical University, Peking University, and local community hospitals and home settings.

4.Expert group: A multidisciplinary team is involved, including clinical experts (sports scientists, exercise medicine professionals, public health graduate students), pulmonologists, nurses, rehabilitation therapists, and psychological counselors.

Below is a draft of the proposed oxygen therapy–based exercise intervention plan for children with asthma, developed through our literature review and expert group discussions. Please evaluate the feasibility and importance of each item using a 5-point Likert scale.

Please rate each item’s feasibility and importance. If you believe certain content is unclear or inappropriate, you may revise or delete it in the “Revision Suggestions” column. If you have additional ideas, please fill in the “Supplementary Items” column. For each item marked as “necessary” or “important,” please do not skip the rating section.

Your expert opinion is extremely valuable. Please carefully assess the entire proposed plan and make your judgments based on its practical significance. Thank you!

**Primary Item: 1. Establish a Multidisciplinary Team**

| Primary Item | Secondary Item | Tertiary Item | Item Importance (5-point scale) | | | | | Item Feasibility (5-point scale) | | | | | Suggested Revisions |
| --- | --- | --- | --- | --- | --- | --- | --- | --- | --- | --- | --- | --- | --- |
|  |  |  | Very Important (5) | Relatively Important (4) | Neutral (3) | Not Very Important (2) | Not Important (1) | Very Feasible (5) | Relatively Feasible (4) | Neutral (3) | Not Very Feasible (2) | Not Feasible (1) |  |
| 1. Establish a Multidisciplinary Team | 1.1 Personnel | 1.1.1 Include project team members such as exercise specialists, graduate students specializing in exercise–medicine integration and public health, respiratory physicians, nurses, rehabilitation therapists, and psychological counselors. |  |  |  |  |  |  |  |  |  |  |  |
|  | 1.2 Responsibilities | 1.2.1 Respiratory physicians and nursing staff provide health education; pediatric exercise experts deliver pre-exercise education to the children and their families; respiratory physicians and pediatric exercise experts conduct pre-exercise assessments for the children; graduate students specializing in exercise–medicine integration and public health assist medical staff in implementing exercise prescriptions during the intervention, ensuring the safety of the children during exercise, monitoring their exercise status, collecting and processing data, and performing other supporting tasks. |  |  |  |  |  |  |  |  |  |  |  |
|  | 1.3 Training | 1.3.1 Physicians and nurses involved in clinical care may incorporate appropriate exercise-assisted interventions into routine treatment and nursing under the guidance of exercise–medicine integration experts. |  |  |  |  |  |  |  |  |  |  |  |
|  |  | 1.3.2 Researchers involved in data collection and analysis must have a thorough understanding of the questionnaire and be able to clearly explain each item in simple terms to the children and their families, in order to collect data as completely and accurately as possible. |  |  |  |  |  |  |  |  |  |  |  |
| 2. Health Education | 2.1 Comprehensive Education | 2.1.1 Respiratory physicians and rehabilitation personnel provide health education, explaining knowledge of respiratory therapy, the potential benefits of exercise for physical and mental health, and offering psychological counseling from a psychological consultant when necessary. |  |  |  |  |  |  |  |  |  |  |  |
|  | 2.2 Pre-Exercise Education | 2.2.1 Pediatric exercise experts explain exercise knowledge and methods in an age-appropriate manner to the children and their caregivers, covering exercise goals, methods, duration, location, safety, and precautions. |  |  |  |  |  |  |  |  |  |  |  |
| 3. Exercise Training | 3.1 Pre-Exercise Assessment | 3.1.1 Respiratory physicians, together with pediatric exercise experts, conduct pre-intervention assessments, evaluating the child’s exercise capacity and health risks to ensure the safety of exercise interventions. The assessment includes respiratory function, physical fitness, exercise ability, and psychological status. |  |  |  |  |  |  |  |  |  |  |  |
|  | 3.2 Exercise Plan Implementation | 3.2.1 Well-controlled asthma  ① Type of exercise: Based on the child’s interests; may include jogging, walking, cycling, etc. Swimming is preferred when conditions permit. ② Exercise intensity: Moderate to high intensity is recommended. ③ Duration and frequency: At least 3–5 times per week, 20–60 minutes per day, with continuous or intermittent sessions. |  |  |  |  |  |  |  |  |  |  |  |
|  |  | 3.2.2 Partially Controlled Asthma  ① Type of exercise: Activities with short durations of physical exertion (<5–10 minutes) and low respiratory load, such as recreational swimming (non-competitive), walking, light jogging, and breathing exercises.  ② Exercise intensity: Recommended to be low to moderate intensity (respiratory rate < 2 times the resting rate; exercise heart rate < 60% of maximum heart rate or <130–140 beats/min).  ③ Duration and frequency: Shorten each exercise session (5–10 minutes); gradually increase duration and intensity based on changes in the patient’s condition and exercise tolerance. Appropriately increase exercise frequency, with intermittent rest, to achieve the training effect through accumulated activity. |  |  |  |  |  |  |  |  |  |  |  |
|  | 3.3 Criteria for Suspending Exercise | 3.3.1 Suspend exercise if heart rate exceeds 70% of the age-predicted maximum heart rate. Maximum heart rate = [(220 − age) − resting heart rate] × (50%–60%) + resting heart rate. |  |  |  |  |  |  |  |  |  |  |  |
|  |  | 3.3.2 Suspend exercise during respiratory infections. |  |  |  |  |  |  |  |  |  |  |  |
|  |  | 3.3.3 Immediately stop exercise if the child experiences symptoms such as dizziness, shortness of breath, excessive sweating, or fatigue, or if the accompanying caregiver observes signs of discomfort or fatigue in the child. |  |  |  |  |  |  |  |  |  |  |  |
|  | 3.4 Exercise Precautions | 3.4.1 Wear a heart rate monitor before each exercise session to assess exertion and dynamically adjust the intensity and volume of exercise to avoid overburdening the child. |  |  |  |  |  |  |  |  |  |  |  |
|  |  | 3.4.2 Choose an appropriate exercise environment; avoid exercising in dry or cold conditions, and avoid allergens or environmental irritants. |  |  |  |  |  |  |  |  |  |  |  |
|  |  | 3.4.3 Perform warm-up exercises; use preventive medication before exercise if necessary. |  |  |  |  |  |  |  |  |  |  |  |
|  |  | 3.4.4 Avoid vigorous activities such as sprinting or fast rope skipping; also avoid exercises requiring prolonged physical exertion (≥10 minutes), such as long-distance running or swimming. |  |  |  |  |  |  |  |  |  |  |  |
| 4.Follow-up Management | 4.1 Follow-up Approach | 4.1.1 A combination of in-person and online follow-up methods will be used. |  |  |  |  |  |  |  |  |  |  |  |
|  | 4.2 Follow-up Implementation | 4.2.1 The follow-up team should consist of interdisciplinary professionals, including physicians, nurses, exercise science experts, and psychological counselors. |  |  |  |  |  |  |  |  |  |  |  |
|  |  | 4.2.2 Follow-up content includes: exercise frequency, exercise intensity/load, Childhood Asthma Control Test (C-ACT) scores, and quality of life assessment scores. |  |  |  |  |  |  |  |  |  |  |  |
|  |  | 4.2.3 Follow-up schedule: the first follow-up will be conducted two weeks after the intervention begins, followed by monthly visits, continuing until six months after the end of treatment. |  |  |  |  |  |  |  |  |  |  |  |
| 5. Outcome Evaluation | 5.1 Compliance Evaluation | 5.1.1 During home-based training after discharge, weekly telephone follow-ups will be conducted. Children and their parents will be encouraged to keep a home diary of training activities, recording the number of times the child meets the weekly exercise goals. Reasons for poor compliance will be inquired, and the intervention plan will be adjusted as needed. |  |  |  |  |  |  |  |  |  |  |  |
|  | 5.2 Effectiveness Evaluation | 5.2.1 Monthly in-person follow-ups will be conducted. The effectiveness of the exercise prescription will be comprehensively evaluated through measures such as Forced Expiratory Volume in 1 second (FEV₁), Forced Vital Capacity (FVC), and Childhood Asthma Control Test (C-ACT) scores. |  |  |  |  |  |  |  |  |  |  |  |
| Additional Items Needed | | |  |  |  |  |  |  |  |  |  |  |  |

**Part Two: Expert Background Information Survey**

1. General Information of the Expert

| Name |  | Research Field |  |
| --- | --- | --- | --- |
| Gender |  | Years of Work Experience |  |
| Age |  | ID Number |  |
| Highest Degree |  | Bank Account for Payment |  |
| Title/Position |  | Contact Number |  |

1. Expert Familiarity and Judgment Basis Survey Form

| Please indicate your level of familiarity with the content of this survey | | | | | | | |
| --- | --- | --- | --- | --- | --- | --- | --- |
| Familiarity Level | Very Familiar | | Familiar | | Somewhat Familiar | | Unfamiliar |
| Self-Evaluation of Expertise |  | |  | |  | |  |
| Basis for Your Judgment | | | | | | | |
| Basis | | Strong | | Moderate | | Weak | |
| Theoretical Analysis | |  | |  | |  | |
| Practical Experience | |  | |  | |  | |
| Peer Understanding | |  | |  | |  | |
| Subjective Judgment | |  | |  | |  | |

Thank you once again for your strong support and assistance. Wishing you good health and a pleasant work life!
