# Supplementary material for: Development and preliminary clinical feasibility of a Delphi-based aerobic exercise prescription for children with asthma
Source: Front Pediatr. 2025 Dec 9;13:1700569. doi: 10.3389/fped.2025.1700569 (PMC12722912; doi:10.3389/fped.2025.1700569)
Supplement: Supplementary file 2 [file Supplementaryfile2.docx]

***Appendix B: Proposed Framework and Preliminary Intervention Plan for Exercise-Based Oxygen Therapy in Children with asthma
Second Round: Expert Consultation Form***

Dear Expert,

Thank you for your strong support and guidance in successfully completing the first round of expert consultation for the project titled “Proposed Framework and Preliminary Intervention Plan for Exercise-Based Oxygen Therapy in Children with asthma.”

Seventeen experts participated in the first round of this study, with 17 valid responses. Based on a summary and analysis of the feedback and suggestions collected from each expert, we compiled the statistical results. An item was considered to have high consensus if its mean importance rating was ≥3.5 with a coefficient of variation <0.25. Items not meeting these criteria or with substantial differences in opinion were revised, removed, or marked in red and gray for expert re-evaluation.

This second-round consultation form includes 20 items related to the proposed plan for exercise-based oxygen therapy for children with asthma. We kindly ask you to rate each item based on its importance and feasibility using a Likert 5-point scale, and provide your feedback in the corresponding columns. If you believe an item is unclear or inappropriate, please provide comments or suggested modifications. If you have additional suggestions, please complete the “Supplementary Items” section. For any item rated as “essential” or “very important,” please do not skip the scoring section.

We would appreciate it if you could return the completed form by July 15.

Thank you once again for your generous support and assistance. Wishing you success in your work and a joyful life.

| Primary Item | Secondary Item | Tertiary Item | Mean Score | Coefficient of Variation | Revisions Based on First-Round Expert Feedback | Item Importance | | | | | Suggested Revisions |
| --- | --- | --- | --- | --- | --- | --- | --- | --- | --- | --- | --- |
| 1. Establish a Multidisciplinary Team | 1.1 Personnel | 1.1.1 Include project team members such as exercise specialists, graduate students specializing in exercise–medicine integration and public health, respiratory physicians, nurses, rehabilitation therapists, and psychological counselors. | 4.76 | 0.44 |  | Very Important (5) | Quite Important (4) | Neutral (3) | Slightly Important (2) | Not Important (1) |  |
|  | 1.2 Responsibilities | **1.2.1** Respiratory physicians and nursing staff provide health education; pediatric exercise experts conduct pre-exercise education for the children and their families; respiratory physicians and pediatric exercise experts perform pre-exercise assessments; graduate students specializing in exercise–medicine integration and public health assist medical staff in implementing exercise prescriptions during the intervention, ensuring the safety of the children during exercise, monitoring their exercise status, collecting and processing data, and performing other supporting tasks. | **4.88** | **0.33** |  |  |  |  |  |  |  |
|  | 1.3 Training | **1.3.1** Led by exercise–medicine integration experts, training will be provided to clinical physicians and nurses involved in routine treatment and care, introducing the detailed implementation plan of exercise-assisted interventions. | **4.76** | **0.44** | One expert suggested clarifying the specific procedures and content of the training. |  |  |  |  |  |  |
|  |  | **1.3.2** Researchers involved in data collection and analysis must have a thorough understanding of the questionnaire and be able to explain each item clearly and in simple terms to the children and their families, in order to collect data as completely and accurately as possible. | **4.71** | **0.47** |  |  |  |  |  |  |  |
| **2. Health Education** | 2.1 Comprehensive Education | **2.1.1** Respiratory physicians and nursing staff provide health education, explaining medical knowledge related to asthma and the positive effects of exercise during the recovery phase. Psychological counselors offer emotional support as needed. Meanwhile, healthcare providers should maintain a positive attitude and communicate promptly with the child’s parents, explaining the child’s condition and progress, and working to build a bridge of trust between medical staff and the family. | **4.65** | **0.49** | Suggestion: Add a component where team members build trust with the child and their parents. |  |  |  |  |  |  |
|  | 2.2 Pre-Exercise Education | **2.2.1** Pediatric exercise experts focus on explaining, in simple and accessible terms, the exercise cycle, the contents of the exercise prescription, the exercise setting, exercise monitoring, and safety considerations to the child and their caregivers. | **4.76** | **0.44** |  |  |  |  |  |  |  |
| 3. Exercise Training | **3.1 Pre-Exercise Assessment** | **3.1.1** The attending physician and pediatric exercise expert will conduct a physical examination to assess exercise tolerance and screen for safety risks in children with asthma who are in the intervention group. This includes an Asthma Control Test (ACT) score, the number of acute episodes in the past month, and baseline pulmonary function (FEV₁/FVC). The child’s exercise capacity will be measured using the 6-Minute Walk Test (6MWT). If the child exhibits symptoms such as respiratory distress during the test, it should be stopped immediately. The exercise intensity for each child will be determined based on their tolerance, physical fitness, and functional movement screening. | **4.76** | **0.44** | One expert suggested adding specific pre-exercise assessment criteria. |  |  |  |  |  |  |
|  | 3.2 Implementation of the Exercise Plan | **3.2.1 Grades 1–2 Group**  **(1) Well-Controlled Asthma Group** ① **Type of exercise**: Based on the child’s interests, select appropriate aerobic activities; swimming is preferred if conditions permit. ② **Exercise intensity**: 45%–65% of maximum heart rate (HRmax). ③ **Duration and frequency**: Twice per week, 30–35 minutes per session.  **(2) Partially Controlled Asthma Group** ① **Type of exercise**: Choose aerobic activities with short durations of physical exertion (<5–10 minutes) and low respiratory load. ② **Exercise intensity**: 40%–55% of HRmax. ③ **Duration and frequency**: Twice per week, 25–30 minutes per session. |  |  | Two experts suggested that the implementation of exercise prescriptions should be differentiated by age group. |  |  |  |  |  |  |
|  |  | **3.2.2 Grades 3–4 Group**  **(1) Well-Controlled Asthma Group** ① **Type of exercise**: Based on the child’s interests, select appropriate aerobic activities; swimming is preferred if conditions permit. ② **Exercise intensity**: 50%–70% of maximum heart rate (HRmax). ③ **Duration and frequency**: Three times per week, 35–40 minutes per session.  **(2) Partially Controlled Asthma Group** ① **Type of exercise**: Choose aerobic activities with short durations of physical exertion (<5–10 minutes) and low respiratory load. ② **Exercise intensity**: 45%–65% of HRmax. ③ **Duration and frequency**: Three times per week, 30–35 minutes per session. |  |  |  |  |  |  |  |  |  |
|  |  | **3.2.3 Grades 5–6 Group**  **(1) Well-Controlled Asthma Group** ① **Type of exercise**: Based on the child’s interests, select appropriate aerobic activities; swimming is preferred if conditions permit. ② **Exercise intensity**: 55%–75% of maximum heart rate (HRmax). ③ **Duration and frequency**: 3–4 times per week, 40–45 minutes per session.  **(2) Partially Controlled Asthma Group** ① **Type of exercise**: Choose aerobic activities with short durations of physical exertion (<5–10 minutes) and low respiratory load. ② **Exercise intensity**: 50%–70% of HRmax. ③ **Duration and frequency**: 3–4 times per week, 40–45 minutes per session. |  |  |  |  |  |  |  |  |  |
|  | 3.3 Criteria for Suspending Exercise | **3.3.1** Exercise should be suspended during acute asthma attacks or respiratory infections. Key symptoms include: sudden onset of frequent coughing, chest tightness, wheezing, and difficulty breathing during exercise, accompanied by a “whistling” sound (wheezing) during exhalation that progressively worsens.(Muscle soreness, fatigue, labored breathing, and chest tightness during exercise are more common in individuals who are obese or have been inactive for a long time. These symptoms are usually related to the accumulation of local metabolic byproducts such as lactic acid. If these symptoms quickly resolve after rest, they are not considered an acute asthma attack and may improve gradually with progressive exercise training.) | **4.88** | **0.33** | Two experts suggested listing specific criteria to distinguish between exercise-induced asthma and general physical fatigue. |  |  |  |  |  |  |
|  |  | **3.3.2** It is recommended to carry a peak flow meter to measure Peak Expiratory Flow (PEF). If the PEF value is less than 80% of the predicted value, the child should use a rescue inhaler or participate in low-intensity activity under observation. After 10 to 20 minutes, retest the PEF. If it returns to normal, regular exercise may be resumed. | **5.00** | **0.00** | Two experts suggested clarifying the criteria and plan for resuming exercise after it has been suspended. |  |  |  |  |  |  |
|  |  | **3.3.3** Physical exercise is not recommended when the child is easily fatigued, emotionally tense, or under significant psychological stress. | **4.71** | **0.59** |  |  |  |  |  |  |  |
|  | 3.4 Exercise Precautions | **3.4.1** Each child will be assigned a heart rate monitor by the hospital (certified for pediatric use). Before each exercise session, the child should wear the monitor to assess physical load. Exercise volume and intensity should be dynamically adjusted to avoid overexertion and ensure the child’s physical tolerance. | **4.76** | **0.09** | Three experts suggested clarifying the distribution method of the heart rate monitors. |  |  |  |  |  |  |
|  |  | **3.4.2** Choose an appropriate exercise environment; avoid exercising in dry or cold conditions, and avoid exposure to allergens and environmental irritants. | **4.71** | **0.12** |  |  |  |  |  |  |  |
|  |  | **3.4.3** Adequate warm-up exercises should be performed before physical activity, mainly consisting of low to moderate intensity exercises. A "fun warm-up module" can be designed—for example, 5 minutes of animal imitation games (for stretching) followed by 5 minutes of slow jump rope—to balance children's interests with safety. The total warm-up duration should be 10–15 minutes (e.g., brisk walking). Preventive medication should be used before exercise when necessary. | **4.82** | **0.08** | One expert suggested adding more detailed content for the warm-up exercises. |  |  |  |  |  |  |
|  |  | **3.4.4** Avoid high-intensity activities such as sprinting and fast jump rope; also avoid exercises involving sustained physical exertion (≥10 minutes), such as long-distance running or long-distance swimming. | **4.82** | **0.08** |  |  |  |  |  |  |  |
| 4. Follow-up Management | 4.1 Follow-up Management Approach | **4.1.1** Includes a combination of in-person (offline) and online follow-up visits. | **4.71** | **0.59** |  |  |  |  |  |  |  |
|  | 4.2 Implementation of Follow-up Management | **4.2.1** The follow-up team should consist of interdisciplinary professionals, including physicians, nurses, exercise science experts, and psychological counselors. | **4.41** | **0.62** | Suggestion: Improve and expand the follow-up content. |  |  |  |  |  |  |
|  |  | **4.2.2** Follow-up content includes: exercise frequency, exercise load, Childhood Asthma Control Test (C-ACT) scores, and quality of life assessments. Team members should regularly monitor the child’s exercise status online and provide professional guidance. | **4.47** | **0.62** |  |  |  |  |  |  |  |
|  |  | **4.2.3** Follow-up schedule: the initial follow-up should be conducted two weeks after the start of the intervention, followed by monthly follow-ups, continuing until three months after the end of treatment. | **4.26** | **0.66** |  |  |  |  |  |  |  |
| 5. Outcome Evaluation | 5.1 Compliance Evaluation | **5.1.1** During home-based training after discharge, weekly telephone follow-ups will be conducted. Children and their parents will be encouraged to use a home diary to record training details, including the average number of times per week the child meets the exercise goals. Reasons for poor compliance will be identified, and the intervention plan will be adjusted as necessary. | **4.59** | **0.62** |  |  |  |  |  |  |  |
|  | 5.2 Effectiveness Evaluation | **5.2.1** Monthly in-person follow-ups will be conducted. The effectiveness of the exercise prescription will be comprehensively evaluated through measures such as Forced Expiratory Volume in 1 second (FEV₁), Forced Vital Capacity (FVC), and Childhood Asthma Control Test (C-ACT) scores. | **4.53** | **0.62** |  |  |  |  |  |  |  |
| Additional Items Needed | |  | | | | | | | | | |
