# Supplementary material for: Development and preliminary clinical feasibility of a Delphi-based aerobic exercise prescription for children with asthma
Source: Front Pediatr. 2025 Dec 9;13:1700569. doi: 10.3389/fped.2025.1700569 (PMC12722912; doi:10.3389/fped.2025.1700569)
Supplement: Supplementary file 3 [file Supplementaryfile3.docx]

Appendix C: Development of an Aerobic Exercise Prescription and Intervention Plan for Children with Asthma Based on the Delphi Method

Final Version of the Expert Consultation Form

| Primary Item | Secondary Item | Tertiary Item | Item Importance (5-point scale) | | | | | Item Feasibility (5-point scale) | | | | | Suggested Revisions |
| --- | --- | --- | --- | --- | --- | --- | --- | --- | --- | --- | --- | --- | --- |
|  |  |  | Very Important (5) | Relatively Important (4) | Neutral (3) | Not Very Important (2) | Not Important (1) | Very Feasible (5) | Relatively Feasible (4) | Neutral (3) | Not Very Feasible (2) | Not Feasible (1) |  |
| 1. Establish a Multidisciplinary Team | 1.1 Personnel | 1.1.1 Include project team members such as exercise specialists, graduate students specializing in exercise–medicine integration and public health, respiratory physicians, nurses, rehabilitation therapists, and psychological counselors. |  |  |  |  |  |  |  |  |  |  |  |
|  | 1.2 Responsibilities | 1.2.1 Respiratory physicians and nursing staff provide health education; pediatric exercise experts deliver pre-exercise education to the children and their families; respiratory physicians and pediatric exercise experts conduct pre-exercise assessments; graduate students specializing in exercise–medicine integration and public health assist medical staff in implementing the exercise prescription during the intervention, ensuring the child’s exercise safety, monitoring their physical condition, collecting and processing data, and performing other supporting tasks. |  |  |  |  |  |  |  |  |  |  |  |
|  | 1.3 Training | 1.3.1 Experts in exercise–medicine integration will lead training sessions for clinical physicians and nurses involved in routine treatment and care, introducing the detailed implementation plan for exercise-assisted interventions. |  |  |  |  |  |  |  |  |  |  |  |
|  |  | 1.3.2 Researchers involved in data collection and analysis must have a thorough understanding of the questionnaire and be able to clearly explain each item in simple terms to the children and their families, in order to collect data as completely and accurately as possible. |  |  |  |  |  |  |  |  |  |  |  |
| 2. Health Education | 2.1 Comprehensive Education | 2.1.1 Respiratory physicians and nursing staff provide health education, explaining medical knowledge about asthma and the positive effects of exercise during the recovery period. They also clarify the mechanisms by which aerobic exercise improves lung function—such as enhancing diaphragmatic strength and reducing inflammatory cytokine levels. Psychological counselors offer emotional support as needed. Meanwhile, healthcare providers should maintain a positive attitude and communicate promptly with the child’s parents, working to build a relationship of trust. This helps families understand the content and implementation of the exercise prescription, enabling parents to assist and supervise their child’s physical activity in daily life. |  |  |  |  |  |  |  |  |  |  |  |
|  | 2.2 Pre-Exercise Education | 2.2.1 Pediatric exercise experts focus on explaining to the child and their family—in clear and simple terms—the purpose of exercise, the exercise cycle, the contents of the exercise prescription, appropriate exercise locations, methods for monitoring exercise, and important precautions. |  |  |  |  |  |  |  |  |  |  |  |
| 3. Exercise Training | 3.1 Pre-Exercise Assessment | 3.1.1 The attending physician and pediatric exercise expert will conduct a physical examination to assess exercise tolerance and screen for safety risks in children with asthma who are in the intervention group. This includes the Asthma Control Test (ACT) score, the number of acute episodes in the past month, and baseline pulmonary function (FEV₁/FVC). The child’s exercise capacity will be evaluated using the 6-Minute Walk Test (6MWT). If the child experiences symptoms such as respiratory distress during the test, it should be terminated immediately. The exercise intensity for each child will be determined based on their tolerance, physical fitness, and results from functional movement screening. |  |  |  |  |  |  |  |  |  |  |  |
|  | 3.2 Implementation of the Exercise Plan | 3.2.1 Grades 1–2 Group  (1) Well-Controlled Asthma Group ① Type of exercise: Select aerobic exercises based on the child’s interests. Swimming is preferred when conditions permit. (Note: swimming pools should be suitable for children with asthma, following “asthma-friendly pool selection criteria,” including chlorine concentration ≤ 0.5 ppm, equipped with an air circulation system, and heated water temperature of 28–30°C.) ② Exercise intensity: 45%–65% of maximum heart rate (HRmax). ③ Duration and frequency: Twice per week, 30–35 minutes per session.  (2) Partially Controlled Asthma Group ① Type of exercise: Select aerobic exercises that involve short bursts of effort (<5–10 minutes) and low respiratory load. ② Exercise intensity: 40%–55% of HRmax. ③ Duration and frequency: Twice per week, 25–30 minutes per session. |  |  |  |  |  |  |  |  |  |  |  |
|  |  | 3.2.2 Grades 3–4 Group  (1) Well-Controlled Asthma Group ① Type of exercise: Select appropriate aerobic activities based on the child’s interests. Swimming is preferred if conditions allow. ② Exercise intensity: 50%–70% of maximum heart rate (HRmax). ③ Duration and frequency: Three times per week, 35–40 minutes per session.  (2) Partially Controlled Asthma Group ① Type of exercise: Choose aerobic exercises that involve short periods of exertion (<5–10 minutes) and low respiratory load. ② Exercise intensity: 45%–65% of HRmax. ③ Duration and frequency: Three times per week, 30–35 minutes per session. |  |  |  |  |  |  |  |  |  |  |  |
|  |  | 3.2.3 Grades 5–6 Group  (1) Well-Controlled Asthma Group ① Type of exercise: Select appropriate aerobic activities based on the child’s interests. Swimming is preferred when conditions permit. ② Exercise intensity: 55%–75% of maximum heart rate (HRmax). ③ Duration and frequency: 3–4 times per week, 40–45 minutes per session.  (2) Partially Controlled Asthma Group ① Type of exercise: Choose aerobic activities that involve short bursts of effort (<5–10 minutes) and low respiratory load. ② Exercise intensity: 50%–70% of HRmax. ③ Duration and frequency: 3–4 times per week, 40–45 minutes per session. |  |  |  |  |  |  |  |  |  |  |  |
|  | 3.3 Criteria for Suspending Exercise | 3.3.1 Exercise should be suspended during an acute asthma attack or respiratory infection. Typical symptoms include sudden onset of frequent coughing, chest tightness, wheezing, and difficulty breathing during exercise, with a progressive worsening trend, and a “whistling” sound (wheezing) heard during exhalation.(Muscle soreness, weakness, labored breathing, and chest tightness during exercise—often observed in obese or sedentary individuals—are typically caused by the accumulation of local metabolic byproducts such as lactic acid. If these symptoms are quickly relieved after rest, they are not considered an acute asthma attack and may be gradually improved through progressive exercise training.) |  |  |  |  |  |  |  |  |  |  |  |
|  |  | 3.3.2 It is recommended to carry a peak flow meter to measure Peak Expiratory Flow (PEF). If the PEF value is less than 80% of the predicted value, the child should either use a rescue inhaler or engage in low-intensity activities under observation. PEF should be retested after 10 to 20 minutes, and if it returns to normal, regular exercise may be resumed. |  |  |  |  |  |  |  |  |  |  |  |
|  |  | 3.3.3 Physical exercise is not recommended when the child is easily fatigued, emotionally tense, or experiencing excessive psychological stress. |  |  |  |  |  |  |  |  |  |  |  |
|  | 3.4 Exercise Precautions | 3.4.1 Each child will be provided with a heart rate monitor by the hospital (certified for pediatric use). The child should wear the monitor before each exercise session to track heart rate. Based on the principle of not overloading the child’s body, the exercise volume and intensity should be dynamically adjusted accordingly. |  |  |  |  |  |  |  |  |  |  |  |
|  |  | 3.4.2 Choose an appropriate exercise environment; avoid exercising in dry or cold conditions, and avoid exposure to allergens and environmental irritants. |  |  |  |  |  |  |  |  |  |  |  |
|  |  | 3.4.3 A thorough warm-up should be performed before exercise, mainly consisting of low- to moderate-intensity activities. A “fun warm-up module” may be designed—for example, 5 minutes of animal imitation games (for stretching) plus 5 minutes of slow jump rope—to balance children's interest and safety. The total warm-up time should be 10–15 minutes (e.g., brisk walking). Preventive medication should be taken before exercise when necessary. |  |  |  |  |  |  |  |  |  |  |  |
|  |  | 3.4.4 Avoid high-intensity activities such as sprinting or fast jump rope. Also avoid exercises that require sustained physical exertion (≥10 minutes), such as long-distance running or long-distance swimming. |  |  |  |  |  |  |  |  |  |  |  |
| 4. Follow-up Management | 4.1 Follow-up Management Approach | 4.1.1 The follow-up includes a combination of in-person (offline) and online visits. |  |  |  |  |  |  |  |  |  |  |  |
|  | 4.2 Implementation of Follow-up Management | 4.2.1 The follow-up team should be composed of interdisciplinary professionals, including physicians, nurses, exercise science experts, and psychological counselors. |  |  |  |  |  |  |  |  |  |  |  |
|  |  | 4.2.2 Follow-up content includes: exercise frequency, exercise load, Childhood Asthma Control Test (C-ACT) scores, and quality of life assessments. Team members should regularly monitor the child’s exercise status online and provide professional guidance. |  |  |  |  |  |  |  |  |  |  |  |
|  |  | 4.2.3 Follow-up schedule: The initial follow-up should take place two weeks after the start of the intervention, followed by monthly follow-ups, continuing until six months after the end of treatment. |  |  |  |  |  |  |  |  |  |  |  |
| 5. Outcome Evaluation | 5.1 Compliance Evaluation | 5.1.1 During home-based training after discharge, weekly telephone follow-ups will be conducted. Children and their parents will be encouraged to use a home diary to record training details, including the average number of times per week the child meets the exercise goals. Reasons for poor compliance will be explored, and the intervention plan will be adjusted if necessary. |  |  |  |  |  |  |  |  |  |  |  |
|  | 5.2 Effectiveness Evaluation | 5.2.1 Monthly in-person follow-ups will be conducted. The effectiveness of the exercise prescription will be comprehensively evaluated based on assessments such as Forced Expiratory Volume in 1 second (FEV₁), Forced Vital Capacity (FVC), and Childhood Asthma Control Test (C-ACT) scores. |  |  |  |  |  |  |  |  |  |  |  |
| Additional Items Needed | | |  | | | | | | | | | | |
