# Supplementary material for: Development and preliminary clinical feasibility of a Delphi-based aerobic exercise prescription for children with asthma
Source: Front Pediatr. 2025 Dec 9;13:1700569. doi: 10.3389/fped.2025.1700569 (PMC12722912; doi:10.3389/fped.2025.1700569)
Supplement: Supplementary file 4 [file Supplementaryfile4.docx]

Attachment D Informed Consent

Patient Information Collection Form

Dear patient：

I would like to thank you for taking the time out of your day to do this study with us. In this study, we will communicate with you through the Intemet and conduct a physical examination to find out your physical condition and tolerance level. We will provide you with a sealed intervention plan, , in order to help you improve your immunity and survival, and not to cause any harm to you, so that you will not have to worry about any harm. This study will be conducted in an anonymous manner, and we will not use any of the information collected for the purpose of the study. The questions are divided into two parts: and You can just answer the questions by answering the questions in your own situation. Thanks again for your cooperation and help!

The newest example of informed consent is:
