# Supplementary material for: Development and preliminary clinical feasibility of a Delphi-based aerobic exercise prescription for children with asthma
Source: Front Pediatr. 2025 Dec 9;13:1700569. doi: 10.3389/fped.2025.1700569 (PMC12722912; doi:10.3389/fped.2025.1700569)
Supplement: Supplementary file 5 [file Supplementaryfile5.docx]

***Appendix E Basic Information***

T101. Gender: 1. Male 2. Female

T102. Height: ________ cm Weight: ________ kg

T103. Age: ____ years old

T104. Place of residence: 1. Urban 2. Rural

T105. Monthly household income: 1. Below 5,000 yuan 2. 5,000 to 8,000 yuan 3. Above 8,000 yuan

T106. Form of medical insurance: (multiple selections allowed)

1. Self-funded 2. Health insurance

T107. Do any of your immediate family members have asthma? 1. Yes 2. No

T108. How long have you had asthma attacks? 1. ≤1 year 2. 2–3 years 3. 4–5 years 4. >5 years

T109. How many times have you been hospitalized for asthma? ____ times; Length of each hospitalization: ____ days

T110. Is air pollution severe in the city where you live? 1. Severe 2. Moderate 3. Mild

T111. What do you think caused your asthma? [Multiple choice] 1. Genetics 2. Allergens 3. Dust mites

4. Air pollution 5. Excessive exercise 6. Other

T112. How many times per week do you engage in regular exercise? 1. 0 times 2. 1–2 times 3. 3–5 times 4. More than 5 times

T113. How long does each exercise session last? 1. Less than half an hour 2. Half an hour to 1 hour 3. 1 hour to 2 hours 4. Over 2 hours

T114. Do you experience difficulty due to asthma while running or exercising?

1. Extremely difficult, unable to exercise at all 2. Often difficult, feeling uncomfortable 3. Some difficulty, but not too bad 4. No difficulty at all

T115. How has asthma affected your studies in the past month?

1. No impact 2. Some impact 3. Significant impact

T116. Your usual sleep duration: 1. Over 10 hours 2. 10–9 hours 3. 9–8 hours 4. 8–7 hours 5. Under 7 hours

T117. How would you rate the quality of your sleep? 1. Very good 2. Fairly good 3. Fairly poor 4. Very poor
