# Supplementary material for: Development and preliminary clinical feasibility of a Delphi-based aerobic exercise prescription for children with asthma
Source: Front Pediatr. 2025 Dec 9;13:1700569. doi: 10.3389/fped.2025.1700569 (PMC12722912; doi:10.3389/fped.2025.1700569)
Supplement: Supplementary file 6 [file Supplementaryfile6.docx]

***Appendix F: Childhood Asthma Control Test (C-ACT Scale)***

This questionnaire consists of 7 questions, with the first 4 questions each having 4 possible answers. Asthmatic children complete these questions independently. If assistance is needed, parents may help the child understand the questions. Asthmatic

The last 3 questions have 5 possible answers and are completed by the child's family members. The total score is calculated by a pediatric professional. A score >23 indicates complete control,

20–22 indicates partial control, and ≤19 indicates uncontrolled asthma.

The 7 questions in this questionnaire are as follows:

T201. How is your asthma today? 0. Very bad 1. Bad 2. Good 3. Very good

T202. How much of a problem is asthma when you run, exercise, or play sports, and how much does it affect you?

0. It's a big problem; I can't do what I want to do 1. It's a problem; I don't like it 2. It's a small problem; I can handle it 3. No problem

T203. Do you cough because of your asthma?

0. Yes, all the time 1. Yes, most of the time 2. Yes, sometimes 3. Never

T204. Do you wake up at night because of your asthma?

0. Yes, all the time 1. Yes, most of the time 2. Yes, sometimes 3. Never

T205. In the past 4 weeks, how many days did your child have daytime asthma symptoms?

0. Every day 1. 19–24 days 2. 11–18 days 3. 4–10 days 4. 1–3 days 5. None

T206. In the past 4 weeks, how many days did your child have wheezing during the day due to asthma?

0. Every day 1. 19–24 days 2. 11–18 days 3. 4–10 days 4. 1–3 days 5. None

T207. In the past 4 weeks, how many days did your child wake up at night due to asthma?

0. Every day 1. 19–24 days 2. 11–18 days 3. 4–10 days 4. 1–3 days 5. None
